# 

**Published:** 1807-09

**Authors:** 


### To CORRESPONDENTS.

We have received Mr. Golding's Answer to Mr. Ring; but as Mr. G. as well as the Correspondent he quotes, have totally mistaken the question, we cannot publish it. They suppose the only doubt to be, whether Eliza had the small-pox; a doubt which, we apprehend, no one entertains. Mr. Ring, on the contrary, asserts that she never was *regularly vaccinated;* an opinion in which, we think, all intelligent Practitioners will agree.

The Observations of Tyro are too sarcastic for publication.

Mr. Marson's Answer to Mr. Ring is received, but we consider that account as closed for the present.

